# Validating 7-items Overactive Bladder Symptom Score (OABSS) through Arabic linguistic version

**DOI:** 10.1038/s41598-020-79974-9

**Published:** 2021-01-12

**Authors:** Fadi Sawaqed, Mohammed Suoub

**Affiliations:** Section of Urology, Department of Special Surgery, Faculty of Medicine, Mut’ah University, Karak, 61710 Jordan

**Keywords:** Diseases, Health care, Medical research, Urology

## Abstract

The scoring of the 7-item Overactive Bladder Symptom Score (OABSS) questionnaire is unusual because its scale varies with the same maximum and minimum scoring values and quantifies all aspects of OAB. The questionnaire also contains a graded response for urgency. The current study is mainly concerned with the development and validation of the OABSS questionnaire for Jordanian patients. The process of translating the English OABSS questionnaire into the Arabic language involved forward and backward translations. Afterward, a prospective study was conducted to validate the Arabic version of the OABSS questionnaire by examining 235 patients from the outpatient clinics of Karak Governorate Teaching Hospital. The Arabic OABSS questionnaire was completed by all the enrolled patients before and after three months of treatment with solifenacin 5 mg taken once daily. The study included 235 regular patients (152 females and 83 males) diagnosed with OAB in accordance with the definition of the International Continence Society (ICS). The results showed major and significant differences on all seven domains of the questions on the OABSS questionnaire before and after receiving treatment (*p* < 0.05). Confirmatory Factor Analysis was used to measure the reliability and the questionnaire was found to be highly reliable for the construct variables. The findings derived from the current study would be beneficial for local urologists and researchers, as the Arabic version of the OABSS questionnaire was proven to be a reliable instrument for use in the assessment of OAB. Future studies are needed to compare different translated questionnaires relating to OAB.

Trial registration number: NCT04309890.

## Introduction

Overactive bladder (OAB) is associated with the urgency to urinate that may or may not be accompanied by urinary continence^1^. In the absence of a urinary tract infection, the urgency of urinary incontinence is associated with nocturia and increased daytime frequency^2^. This condition has severe adverse effects on the physical and social functioning of an individual, including sleep patterns, work life, and social relationships^3^. There is a negative impact of urge incontinence on the health-related quality of life in patients suffering from OAB^3^. Patients suffering from this condition experience a low quality of life compared to community controls, even though they are not suffering from incontinence^4^. Studies have shown a positive relationship between the severity of OAB symptoms and the experience of symptoms^4,5^. The main symptoms that bother most patients are urgency, nocturia, and urge incontinence.

Elbaset et al.^6^ reported that urination frequency and the occurrence of urgency and urge urinary incontinence (UUI) are predominant in OAB cases: 85%, 54%, and 36%, respectively. Previous studies have reported an increased prevalence of OAB in European patients (11.8%)^7,8^. This condition causes an adverse impact on an individual’s life^9^. It has been observed that the prevalence of OAB increases from 12 to 20% with respect to an increase in an individual’s age^10^.

Urine leakage and urge incontinence severely bother most patients and adversely affect many aspects of their life, such as their emotional well-being, productivity at work, daily activities, social relationships, and personal relationships^4^. Robinson^11^ demonstrated that anticholinergic medications, physiotherapy, bladder training, and electrical stimulation are treatments for OAB. The other treatment options include behavioural therapy, intravesical injection of botulinum toxin, lifestyle modification, pharmacotherapy, and surgical procedures. The surgical procedures include neuromodulation and bladder augmentation. Maximal comfort in patients with OAB with minimal side effects can be accomplished through flexible dosing of anticholinergic drugs^12^.

The assessment of OAB is critical for patients as well as physicians who are concerned with the evaluation of the effectiveness of treatment. Its diagnosis is easily carried out based on the symptoms^3^. The impact of OAB and its treatment outcomes need to be assessed through multi-item questionnaires. Generally, women are most affected by overactive bladder, but they hesitate to discuss such complications with medical experts or specialists, which complicates the condition^13^.

Reflecting upon the treatment objectives, all these outcomes are directed towards overcoming OAB symptoms^14^. However, the efficacy of the treatment is judged mainly by the patient’s response. The resistance and hesitation of patients in reporting the symptoms, which they often neglect during treatment, make the condition severe^15^. Therefore, a self-report questionnaire is the most suitable tool for evaluating the perspectives of patients concerning OAB. The purpose of the questionnaire varies with its use^1^. These practices are not only limited to assessing patients’ perspectives but are also used for monitoring the treatment progress or for a post-treatment evaluation. The validity and objectivity of the questionnaire for assessing the symptoms of OAB and its impact on quality of life were found by the development of the Overactive Bladder Symptom Score (OABSS) questionnaire^16^.

Blaivas et al.^17^ developed the OABSS questionnaire to resolve the concerns of physicians who provide treatment to patients suffering from OAB. This questionnaire tends to employ a self-administered questionnaire for quantifying the symptoms of OAB. This questionnaire covers four symptoms experienced by patients: urgency incontinence, urgency, daytime frequency, and night-time frequency; it also quantifies all aspects of OAB, including graded response for urgency^18^. The scoring in this questionnaire is unusual, as its scale varies with the same maximum and minimum scoring values (score, OABSS). Moreover, Shim et al.^18^, has also identified that the primary concern of physicians regarding the use of the OABSS questionnaire in treatment is the lack of acceptance of its questions for assessing OAB. It is important to have the OABSS questionnaire tested for its reliability and validity to adequately measure OAB symptoms. This will provide verification and confidence for its usage in clinical assessment.

The significant interest of the study regarding the OABSS is its development and validation for its promising usage in Jordanian patients; therefore, it also involves Arabic translation. The psychometric symptoms inquired about in the questionnaire are significantly influenced by the cultural background of patients. Therefore, the current study addresses the development and validation of the Arabic version of the OABSS questionnaire for Jordanian patients. This Arabic translation of the questionnaire helps in assessing patients’ perspectives and monitoring their treatment progress. It could also be used for post-treatment evaluation. Ultimately, it will help in conducting more research on OAB in Jordan and other Arabic countries where there is a literature gap on OAB.

## Materials and methods

### Ethics

The procedures followed in this study wholly complied with the ethical standards of the Responsible Committee on Human Experimentation (Karak Governorate Teaching Hospital) and with the Helsinki Declaration of 1975, which was revised in 2000. The study was approved by the Institutional Ethical Committee of the Faculty of Medicine, Mu’tah University. Informed consent was also obtained from patients. Moreover, patients’ personal information, such as names, initials, and hospital numbers, was kept confidential.

### Study design

#### Overview of the selection and description of participants

This prospective study validated the symptoms of OAB through the OABSS questionnaire, following the hospital’s protocol. A total of 235 patients from urology outpatient clinics of Karak Governorate Teaching Hospital who met the criteria described in the International Continence Society’s definition of OAB were purposively enrolled in this study. The following are the inclusion criteria: aged more than 20 years and three-month history of OAB symptoms (urgency with or without incontinence and associated frequency). Patients were excluded from the study if they had an active urinary tract infection, predominant symptoms of urinary stress incontinence, diabetes mellitus, previous abdominal and pelvic (urological, gynaecological or gastrointestinal) surgery, or a previous history of neurological disease.

#### Technical information

Permission to use the OABSS questionnaire (see Online Appendix) was obtained from Blaivas et al.^17^ via email. The scoring in this questionnaire is unusual because its scale varies with the same maximum and minimum scoring values. The English version of the OABSS questionnaire was initially translated by the author whose first language is Arabic, but he received both undergraduate and postgraduate medical education in English and had clinical fellowship training in English in Canada. An expert panel was formulated to evaluate the first version of the translation, and all members of the panel spoke Arabic as their first language. These individuals also received undergraduate and postgraduate medical education in English and had clinical fellowship training in English in Canada and the United States of America. The panel members reviewed and compared the original questionnaire and first Arabic version with respect to clarity, content relevance, and suitability to the Jordanian community.

The first version was edited according to the recommendations of the panel of experts, and a second version was created. Finally, the questionnaire was back-translated by a translator specialized in medical translation, and a comparison was made between the original and the back-translated versions, which were almost identical at this stage. The questionnaire was validated by comparing the responses to the questionnaire from a patient with OAB before and after treatment with antimuscarinics.

All enrolled patients were given to self-complete the Arabic OABSS questionnaire in its final edition. Each participant filled out the questionnaire twice, before the pharmacological treatment and three months after their treatment with solifenacin 5 mg taken once daily. The OABSS questionnaire comprising seven questions was self-administered to all the patients based on a 5-point Likert rating scale. A pilot test was carried out to evaluate the clarity and appropriateness of the questionnaire by interviewing ten male and ten female patients who may or may not have had OAB symptoms. The interviewed patients described the questions’ simplicity, clarity, and comprehensiveness. No complexities were reported in conducting the pilot test; therefore, no additional changes were made to the questionnaire. The test reliability of the Arabic OABSS questionnaire was moderate to good. Five questions were related to urinary urgency, and two were related to daytime and night-time urinary frequency.

### Statistics of the study

**Figure 1:**
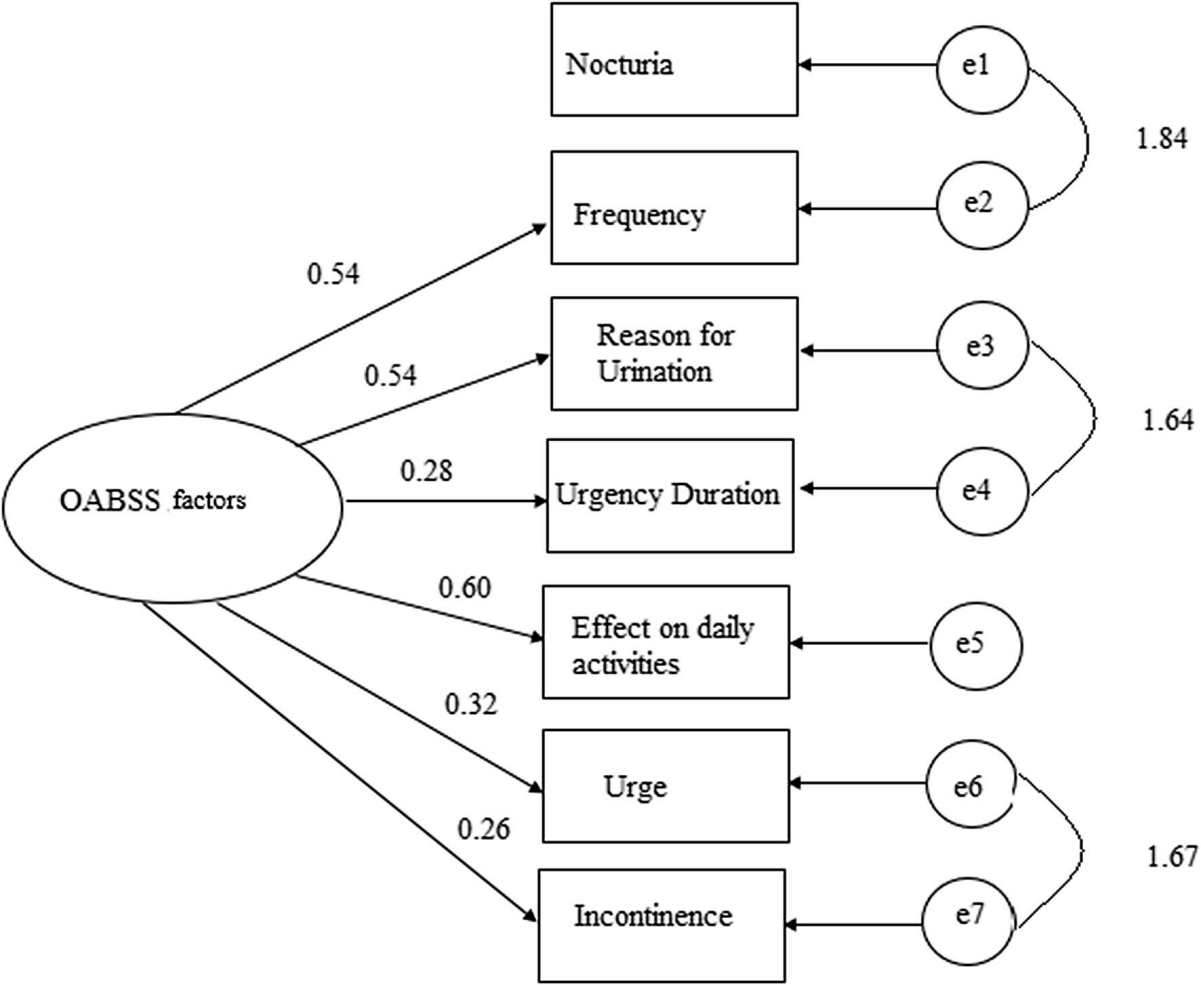
Factor analysis.

The data obtained through the questionnaire were analysed using the IBM Statistical Package of Social Sciences (SPSS) version 20.0. Descriptive statistics were computed for the demographic characteristics of the participants. The study also computed the scoring of frequencies for the seven questions on the questionnaire: daytime frequency (Q1), nocturia (Q2), reason for urination (Q3), postponement duration (Q4), effect of urgency on daily activities (Q5), urgency incontinence (Q6), and bladder control (Q7) subsequent to treatment with solifenacin (before and after). Spearman correlation was used to examine the correlation between the symptoms of OAB in patients. The confirmatory Factor Analysis was done in order to “confirm” the structure model obtained compared to the original version structure. There was strong relationship found between the observed variables and their underlying latent constructs. Therefore, the variables are predictable and the questionnaire is reliable to be used. Intercorrelations were also found between nocturia and frequency, reason for urination and urgency duration, and urge and incontinence (Fig. 1).

### Ethics approval

The study was approved by the Institutional Ethical Committee at the Faculty of Medicine, Mu’tah University.

### Consent to participate

Informed consent was obtained from patients. Their personal information, such as names, initials, and hospital numbers, was kept confidential.

## Results

The study was generated from 235 patients, of which 152 (64.8%) were female and 83 (35.2%) were male (Table 1). Most patients belonged to the age category of 35–49 years (48.5%), and the fewest patients belonged to the age category of 64–78 years (2.1%).

**Table 1:** Demographics.

	Frequency	Percent
**Gender**		
Female	152	64.8
Male	83	35.2
**Age group**		
20–34	57	24.3
35–49	114	48.5
50–63	59	25.1
64–78	5	2.1

Scoring frequencies for the questions on the questionnaire before and after treatment with solifenacin are presented in Table 2. The comparison for all the symptoms in patients before and after treatment with solifenacin was statistically significant. Patients treated with solifenacin showed improved scoring in all domains (*p* < 0.05).

**Table 2:** Scoring for urine frequency before and after treatment with solifenacin.

	Frequency		*P* value
	Before	After	
**Frequency (Q1)**			
No more often than once in 4 h	58 (24.7%)	113 (48.0%)	0.016
About every 3–4 h	111 (47.3%)	113 (48.0%)	
About every 2–3 h	27 (11.5%)	7 (2.1%)	
About every 1–2 h	16 (6.8%)	2 (1.9%)	
At least once an hour	23 (9.7%)	0 (0%)	
**Nocturia (Q2)**			
0–1 times	59 (25.1%)	118 (50.2%)	0.039
2 times	112 (47.7%)	75 (31.9%)	
3 times	31 (13.2%)	42 (17.9%)	
4 times	22 (9.4%)	0 (0%)	
5 or more times	11 (4.6%)	0 (0%)	
**Reason for urination (Q3)**			
No urge or desire	0 (0%)	3 (1.3%)	0.004
Because I have a mild urge or desire	110 (46.8%)	119 (50.6%)	
Because I have a moderate urge or desire	112 (47.6%)	110 (46.8%)	
Because I have a severe urge or desire	9 (3.8%)	2 (0.9%)	
Because I have desperate urge or desire	4 (1.8%)	1 (0.4%)	
**Postponement duration (Q4)**			
More than 60 min	0 (0%)	0 (0%)	0.020
30–60 min	112 (47.6%)	7 (3.0%)	
10–30 min	112 (47.6%)	117 (49.8%)	
A few minutes	8 (3.5%)	110 (46.8%)	
Must go immediately	3 (1.3%)	1 (0.4%)	
**Effect on daily activities (Q5)**			
Never	0 (0%)	116 (49.4%)	0.022
Rarely	1 (0.4%)	116 (49.4%)	
A few times a month	111 (47.2%)	2 (0.8%)	
A few times a week	116 (49.4%)	1 (0.4%)	
At least once a day	7 (2.9%)	0 (0.0%)	
**Urge incontinence (Q6)**			
Never	113 (48.0%)	118 (50.21%)	0.018
Rarely	57 (24.1%)	112 (47.66%)	
A few times a month	48 (20.37%)	4 (1.70%)	
A few times a week	17 (7.23%)	1 (0.42%)	
At least once a day	0 (0%)	0 (0%)	
**Bladder control (Q7)**			
Perfect control	11 (4.68%)	95 (40.4%)	0.045
Very good	91 (38.7%)	117 (49.8%)	
Good	112 (47.6%)	23 (9.78%)	
Limited	17 (7.23%)	0 (0%)	
No control at all	4 (1.70%)	0 (0%)	

Table 3 presents a Pearson correlation of OAB symptoms. The findings showed that there was a positive and significant correlation within the variables at the 5% level of significance (*p* < 0.05). Daytime frequency (Q1) was statistically significantly correlated with nocturia (Q2) (*p* = 0.004), reason for urination (Q3) (*p* = 0.003), and urgency duration (Q4) (*p*, 0.000). A similar statistically significant correlation was found between daytime frequency (Q1) and effect on daily activities (Q5) (*p* = 0.006), urge incontinence (Q6) (*p* = 0.000), and bladder control (Q7) (*p* = 0.002).

**Table 3:** Pearson correlation between overactive bladder symptoms.

		Frequency	Nocturia	Reason for urination	Urgency duration	Effect on daily activities	Urge incontinence	Bladder control
Frequency	Correlation Coefficient	1.000						
	*P* value							
Nocturia	Correlation Coefficient	.474**	1.000					
	*P* value	.004						
Reason for urination	Correlation Coefficient	.491**	.439**	1.000				
	*P* value	.003	.008					
Urgency Duration	Correlation Coefficient	.582**	.448**	.650**	1.000			
	*P* value	.000	.007	.000				
Effect on Daily Activities	Correlation Coefficient	.453**	.605**	.516**	.436**	1.000		
	*P* value	.006	.000	.002	.009			
Urge Incontinence	Correlation Coefficient	.716**	.511**	.591**	.602**	.575**	1.000	
	*P* value	.000	.002	.000	.000	.000		
Bladder Control	Correlation Coefficient	.506**	.405*	.423*	.578**	.424*	.772**	1.000
	*P* value	.002	.016	.011	.000	.011	.000	

**Correlation is significant at the 0.05 level (2-tailed).

For nocturia, a significant and positive correlation was reported for reason for urination (*p* = 0.008) and urgency duration (*p* = 0.007) with OAB symptoms. A similar correlation involving nocturia was observed for effect on daily activities (*p* = 0.000), urge incontinence (*p* = 0.002), and bladder control (*p* = 0.016).

The correlation results were similar for reason for urination, as the obtained p-value was significant for urgency duration (*p* = 0.007), effect on daily activities (*p* = 0.002), and urge incontinence (*p* = 0.000) as well as bladder control (*p* = 0.011). Likewise, urgency duration showed a positive relationship with effect on daily activities (*p* = 0.009), urge incontinence (*p* = 0.000), and bladder control (*p* = 0.000). The correlation of effect on daily activities in patients with OAB was significant for urge incontinence (*p* = 0.000) and bladder control (*p* = 0.011).

## Discussion

The current study was concerned with the development and validation of the OABSS questionnaire in Jordanian patients following Arabic translation. Clinicians use questionnaires to evaluate patients’ symptoms and develop effective treatment strategies. The restricted availability of trained commentators was a challenge for patients and clinicians with respect to treatment and assessment^19^.

The cultural background of the patients significantly affects quesionnaires’ psychometric properties^20,21^. The high and intermediate internal consistency, along with satisfactory interdomain correlations, confirmed the reliability of the Arabic version of the questionnaire. In the current study, the test reliability of the Arabic OABSS questionnaire was moderate to good. Moreover, these results were supported by Italian, Spanish, and Korean linguistic validation studies^22,23^. The validity of the questionnaire was tested for high discriminatory power and good psychometric properties. It was also dependent on the multilevel approach used in the translation process^22,23^.

Symptoms found in male and female patients were mainly related to their quality of life, particularly patients with urinary disorders. The questionnaire used in the current study measured the impact of urge incontinence and symptoms associated with OAB. The symptoms of urinal frequency, nocturia, reason for urination, postponement duration, urge incontinence, bladder control, and their effects on daily living were all correlated, suggesting that they are strongly associated. This shows that the higher the frequency of urination or lesser the control of the bladder is, the higher the number of times these affect activities of daily living. Similarly, less control of the bladder will lead to nocturia. These well-established symptoms of OAB were also found to be associated in this study, suggesting that it is a reliable tool to be used.

Moreover, this type of questionnaire was associated with the health and quality of life of the individuals in new cultures and languages, depending on their destination. Matza et al.^24^ and Homma et al.^25^ examined 47 patients suffering from OAB and showed good reliability. The questionnaire can be used as a discriminatory point for patients suffering from different degrees of nocturia, as it evaluates the impact of nocturia on the quality of life of patients in association with their health and sleep patterns^17^.

The OABSS questionnaire evaluated all the symptoms of OAB, along with a graded response for urgency to be used as a symptom score. The worst symptom graded was severity of urgency^16^. The domains associated with the OABSS questionnaire tend to dilute the efficacy parameters when they are combined. Therefore, it was preferable for these efficacy parameters to be administered as separate outcome instruments^26^. The severity of symptoms and objective measures did not show a strong association; therefore, the total symptom score was likely to be low if an individual was suffering from severe incontinence but not bothered by it^16^. This has justified the different incontinence outcome scores in individuals with similar frequency and amount of incontinence.

This Arabic translation was compared with the Chinese OABSS questionnaire. Chou et al.^27^ validated the Chinese OABSS questionnaire as a reliable instrument to assess OAB. The results showed improvement in urgency, frequency of urination, and urge incontinence after the administration of solifenacin 5 mg on a once-daily basis. These results were consistent with the results of the current study, showing a significant reduction in adverse effects after providing treatment for three months^27^. There are several significant implications of this questionnaire, as it plays an essential role in quantifying OAB symptoms^28^. The questionnaire also permitted graded responses to evaluate urgency, unlike other popular scoring systems concerned with the characterization of other instruments. This questionnaire can help provide a detailed evaluation of urgency symptoms.

The outcomes of the current study reveal that the Arabic version of the OABSS questionnaire is valid and can be easily adopted by clinicians for assessing patients without the help of an interpreter^27^. This Arabic version also decreases shyness and hesitancy in patients, as they can understand it fully without an interpreter. It will also facilitate clinicians’ educating patients about OAB symptoms, its grades, and severity for all age groups. Consequently, it will help in comprehensively understanding the prevalence of OAB in the region and analysing the growing trend of underreported cases in OAB patients. Most importantly, this Arabic version of the questionnaire has a promising role in the clinical care of patients, as it will give urologists and treating physicians a more objective tool in the diagnosis and follow-up of patients with OAB.

Accordingly, urologists should adopt an individualized strategy for treating patients, optimizing treatment, and reducing the detrimental effects experienced prior to treatment. These effects are initially observed in the form of changes in lifestyle and behavioural therapies^29^. Educational programmes to promote awareness of OAB symptoms as well as chronic failure must be conducted to accomplish realistic expectations of treatment^30^. Consequently, treatment objectives must be strategized and individually tailored to address patients’ cases and specific conditions. There is a need to formulate local practising standards in accordance with the domestic conditions and cases to make treatment more applicable and relevant; however, international guidelines are also available for the treatment of patients.

### Limitations

The inclusion of both gender groups with different associated symptoms was a strength of this study. The age differencesin the sample also add to the efficiency of the study. However, the study is limited, as data were collected from only one institute (Karak Governorate Teaching Hospital). The use of the Arabic version of the OABSS questionnaire remains limited in terms of its generalizability because the data were not gathered from different Arabic-speaking regions. The difference in speaking and tongue variation may have also altered the meaning of the questions.

## Conclusion

The current study assessed the Arabic translation involved in the development and validation of the OABSS questionnaire for Jordanian patients. The current study considered the questionnaire to be a useful instrument to quantify the symptoms and outcomes after providing treatment for OAB. The results showed a significant positive difference in urine frequency and reason for urination in patients when treated with solifenacin 5 mg. The greatest improvement was seen in how it affects activities of daily living, as they were positively improved after treatment with solifenacin 5 mg. The findings imply that the patients controlled their habit of postponing urination. All the symptoms of OAB showed a strong association with each other. This questionnaire can be used in adult patients of all genders reliably, as it was easily comprehendible and understandable without an interpreter. The findings derived from the current study would be beneficial for local researchers, as the Arabic Version of the OABSS questionnaire was used in the assessment of OAB and clinical practices in domestic patients.

## Supplementary Information

Supplementary Information.

## Data Availability

The data will be available for review from the corresponding author upon request.
