# Supplementary material for: Validating 7-items Overactive Bladder Symptom Score (OABSS) through Arabic linguistic version
Source: Sci Rep. 2021 Jan 12;11:661. doi: 10.1038/s41598-020-79974-9 (PMC7804958; doi:10.1038/s41598-020-79974-9)
Supplement: Supplementary file 1 — Supplementary Information. [file 41598_2020_79974_MOESM1_ESM.docx]

**Validating 7-items Overactive Bladder Symptom Score (OABSS) through Arabic linguistic version**

**Fadi Sawaqed^1^, Mohammed Suoub^2^**

1-    Assistant Professor of Urology, Section of Urology, Department of Special Surgery, Faculty of Medicine, Mut’ah University, Karak, 61710, Jordan.
[https://orcid.org/0000-0002-5743-880X](https://orcid.org/0000-0002-5743-880X)

2-    Associate Professor of Urology, Section of Urology, Department of Special Surgery, Faculty of Medicine, Mut’ah University, Karak, 61710, Jordan.

<https://orcid.org/0000-0002-6736-0946>

**Appendix:**

**Questionnaire**

1. How often do you usually urinate during the day?

- no more often than once in 4 hours
- about every 3-4 hours
- about every 2-3 hours
- about every 1-2 hours
- at least once an hour

2. How many times do you usually urinate at night (from the time you go to bed until the time you wake up for the day)?

- 0-1 times
- 2 times
- 3 times
- 4 times
- 5 or more times

3. What is the reason that you usually urinate?

- Out of convenience (no urge or desire)
- Because I have a mild urge or desire (but can delay urination for over an hour if I have to)
- Because I have a moderate urge or desire (but can delay urination for more than 10 but less than 60 minutes if I have to)
- Because I have a severe urge or desire (but can delay urination for less than 10 minutes if I have to)
- Because I have desperate urge or desire (must stop what I am doing and go immediately)

4. Once you get the urge or desire to urinate, how long can you usually postpone it comfortably?

- More than 60 minutes
- 30 to 60 minutes
- 10 to 30 minutes
- A few minutes (less than 10 minutes)
- Must go immediately

5. How often do you get a sudden urge or desire to urinate that makes you want to stop what you are doing and rush to the bathroom?

- Never
- Rarely
- A few times a month
- A few times a week
- At least once a day

6. How often do you get a sudden urge or desire to urinate that makes you want to stop what you are doing and rush to the bathroom but you do not get there in time (i.e. you leak urine or wet pads)?

- Never
- Rarely
- A few times a month
- A few times a week
- At least once a day

7. In your opinion how good is your bladder control?

- Perfect control
- Very good
- Good
- Limited
- No control at all
